# Biosynthesis of Anthocyanins and Their Regulation in Colored Grapes

**DOI:** 10.3390/molecules15129057

**Published:** 2010-12-09

**Authors:** Fei He, Lin Mu, Guo-Liang Yan, Na-Na Liang, Qiu-Hong Pan, Jun Wang, Malcolm J. Reeves, Chang-Qing Duan

**Affiliations:** 1 Center for Viticulture and Enology, College of Food Science & Nutritional Engineering, China Agricultural University, Beijing 100083, China; 2 Faculty of Applied Science, Business and Computing, Eastern Institute of Technology, Napier 4142, New Zealand

**Keywords:** anthocyanins, grape, biosynthesis, regulation, phytohormone, environmental factors, viticulture

## Abstract

Anthocyanins, synthesized via the flavonoid pathway, are a class of crucial phenolic compounds which are fundamentally responsible for the red color of grapes and wines. As the most important natural colorants in grapes and their products, anthocyanins are also widely studied for their numerous beneficial effects on human health. In recent years, the biosynthetic pathway of anthocyanins in grapes has been thoroughly investigated. Their intracellular transportation and accumulation have also been further clarified. Additionally, the genetic mechanism regulating their biosynthesis and the phytohormone influences on them are better understood. Furthermore, due to their importance in the quality of wine grapes, the effects of the environmental factors and viticulture practices on anthocyanin accumulation are being investigated increasingly. The present paper summarizes both the basic information and the most recent advances in the study of the anthocyanin biosynthesis in red grapes, emphasizing their gene structure, the transcriptional factors and the diverse exterior regulation factors.

## 1. Introduction

Anthocyanins, synthesized via the flavonoid pathway, have long sparked the interest of biologists, chemists and enologists for they contribute almost all of the orange, pink, red, blue and purple color to colored grapes, their wines and other products [1,2,3,4,5]. Besides their aesthetic values, anthocyanins can offer slight astringency to the mouth feel of the grapes or their products, and can also interact with some aroma substances [6,7,8]. As one of the most ubiquitous class of flavonoids in grape berries, anthocyanins possess a multitude of biological roles, including protection against solar exposure and ultraviolet radiation, free radical scavenging and anti-oxidative capacity, defense against many different pathogens, attraction of predators for seed dispersal, as well as the new proposed modulation of signaling cascades [9,10,11]. Although some researchers have cast doubts on their bioavailability, in recent years considerable attention has been focused on the potential beneficial effects on human health of anthocyanins and their derived compounds in grapes and wines. Such benefits include free radical scavenging and antioxidant activity, antimicrobial and antiviral activity, prevention of cardiovascular disease, protective effect against hepatic damage and disease, anticancer and antimutagenic activity, and so on [12,13,14,15,16,17,18,19,20,21]. On the other hand, with no literature reports of anthocyanin toxicity their safety has been extensively demonstrated by the widespread consumption of food products that contain anthocyanins [22]. Because of their brilliant color, high water solubility and beneficial biological properties, anthocyanins are considered as a class of potential natural pigments to replace the synthetic colorants in many kinds of food [23].

With nearly 70 million tones currently produced worldwide grapes are possibly the world’s largest cultivated fruit, most of which are used in wine making, while the rest are consumed as table grapes or processed into raisins, juices, jams or other products. Of all grapes, cultivars of the *Vitis vinifera* L. species are the most important throughout world, but especially in Europe [1]. Other important grape cultivars belong to *V. rotundifolia*, *V. labrusca*, *V. coignetiae*, *V. rupestris*, *V. amurensis* species and their hybrids with *V. vinifera* or with each other. There are dozens of other less important species of grapes that belong to *Vitis* genus [24,25,26,27,28,29]. Among all of the red cultivars of these grapes, anthocyanins are the main and fundamental colorants. Nevertheless, the amount and composition of anthocyanins present in them varies greatly depending on species, variety, maturity, vintage, region of cultivation and many other factors [30].

Until the middle of the last century, paper chromatography was the preferred analytical method used by most winemakers and enologists to separate anthocyanins in grapes or wines. This was really cumbersome and time-consuming. The development of thin-layer chromatography using silica gel partially speeded the analysis, but this was still a difficult method [31]. In the following several decades, the continuous development of chromatography technologies has greatly facilitated the analysis of anthocyanins in grapes and wines. With the help of modern chromatography methods, such as high-performance liquid chromatography (HPLC) and high-speed countercurrent chromatography (HSCCC), anthocyanins can be separated and quickly and accurately quantified [32,33,34,35]. Moreover qualitative technologies, such as mass spectrometry (MS) and nuclear magnetic resonance (NMR), have enabled anthocyanins to be deconstructed and identified completely and correctly [36,37,38,39]. Another analytical method, capillary electrophoresis (CE), has proved to be an advantageous alternative to HPLC in the anthocyanin analysis in terms of separation efficiency, time required and reagent usage [40,41]. Visible-near-infrared spectrometry (Vis-NIR), has enabled the total anthocyanin concentration of grapes to be predicted quickly. Further development of this method may allow this analysis without the destruction of the grape berries or the wine bottles, which would arouse great interest in grape growers and wine makers, to whom the detailed anthocyanin profiles of the samples is typically unnecessary, while the total content or other basic information can assist with viticultural management practices and harvest timing decisions [42].

Modern instruments and techniques not only facilitate the analysis of anthocyanins in grapes, but also promote the identification and characterization of the key enzymes in the anthocyanin biosynthesis. Although the biosynthesis of anthocyanins in grapes is rather clearer than that of proanthocyanidins (condensed tannins), there is still much about the *in vivo* formation of anthocyanins need to be investigated [43,44,45]. Recently, some novel key enzymes in anthocyanin biosynthesis, some putative anthocyanin transporters and some related transcriptional factors of the MYB family were reported and characterized for grape berries. This has substantially increased our knowledge of anthocyanin biosynthesis, transportation and regulation. In the present review, we summarize the basic knowledge and the new findings in the research field of anthocyanin biosynthesis in grapes.

## 2. Structural Diversity of Anthocyanins in Red Grapes

### 2.1. Individual Anthocyanins

Structurally, anthocyanins are glycosides and acylglycosides of anthocyanidins, and the aglycones, flavyliums (2-phenylbenzopyrilium) differ in the different hydroxyl or methoxyl substitutions in their basic structures [1,27,46]. The core of the anthocyanidin, the flavylium, has the typical C6-C3-C6 flavonoid skeleton, which contains one heterocyclic benzopyran ring (as the C ring), one fused aromatic ring (as the A ring) and one phenyl constituent (as the B ring). In the cation form, anthocyanidins have two double bonds in the C ring and hence carry a positive charge [1,47]. 

The common anthocyanidins found in grapes are pelargonidin (3,5,7,4’-tetrahydroxyflavium), cyanidin (3,5,7,3’,4’-pentahydroxyflavylium), delphinidin (3,5,7,3’,4’,5’-hexahydroxyflavylium), peonidin (3,5,7,4’-tetrahydroxy-3’-methoxyflavylium), petunidin (3,5,7,3’,4’-pentahydroxy-5’- methoxy-flavylium), and malvidin (3,5,7,4’-tetrahydroxy-3’,5’-dimethoxyflavylium) which is usually the predominant anthocyanidin in most red grapes [1,4]. The proportion and amount of each anthocyanidin is influenced greatly by cultivar type and viticultural conditions. This obviously influences both the hue and the color stability, which are directly affected by the hydroxylation and methylation pattern of the B ring of the anthocyanidins. Blueness is enhanced with the increasing of free hydroxyl groups, whereas redness intensifies with the raising of the methylation of the hydroxyl groups as indicated by peak wavelengths (Table 1). Thus, malvidin is also the reddest individual anthocyanidin [48]. On the other hand, the adjacent hydroxyl groups of *o*-diphenols are more sensitive to enzymatic (except for laccase) and nonenzymatic (catalyzed by copper or iron ions) oxidation to produce *o*-diquinones, or even *o*-diphenol dimmers. Therefore, cyanidin, delphinidin and petunidin which contain the *o*-diphenol structure on the B ring are more sensitive to oxidation. On the contrary, neither malvidin nor peonidin possess the *ortho*-positioned hydroxyl groups, which result in their comparatively higher resistance to oxidation [48]. However, some different substitution patterns are also naturally occurring in many other plants, such as 3-deoxyanthocyanidins, 6-hydroxy-anthocyanidins, 5-methoxycyanthocyanidins, and 7-methoxycyanthocyanidins [49].

**Figure 1:**
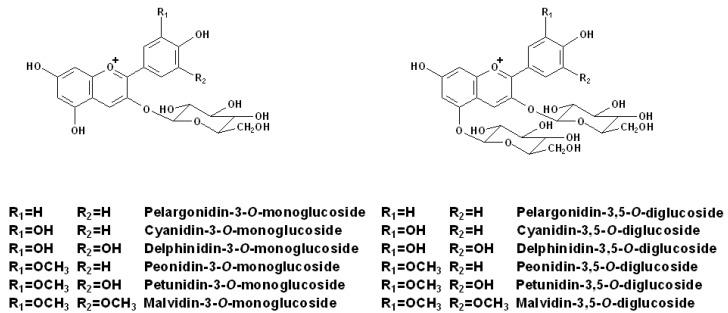
Structures of the individual anthocyanins in grapes [1,48,49].

In most plants, only *O*-glycosylation occurs for anthocyanins. However, some recent literature has also reported the existence of *C*-glycosylation in several plants [50]. In grapes, the sugar moiety is typically glucose. In *V. vinifera* species, the glucose molecules can only be linked to these anthocyanidins through glycosidic bonds at the C_3_ positions to form the 3-*O*-monoglycoside anthocyanins, because they lack the dominant allele involving in the production of diglucosidic anthocyanins [51,52]. In non-*V. vinifera* species, the junctions can occur at both C_3_ and C_5_ positions to produce 3,5-*O*-diglycosides anthocyanins [52]. Thus, the presence of diglucosidic anthocyanins can be normally used to detect the usage of interspecific hybrid grapes in Appellation Control (AC) red wines, because most red French-American hybrids synthesize diglucosidic anthocyanins. Diglucosidic anthocyanins seem to be more stable than their monoglucosidic counterparts, whereas monoglucosidic anthocyanins tend to possess deeper color than their diglucosidic forms [48]. The structures of the individual anthocyanins in grapes, including both anthocyanidin 3-*O*-monoglucosides and 3,5-*O*-diglucosides are illustrated in Figure 1. 

Thus, the structures of anthocyanins are numerous in Nature, and the anthocyanin profile for any kind of plant is distinctive. So far more than 600 different individual anthocyanins have been identified from plants, and this number is still increasing [1,20,49,53]. This is partly due to the fact that in the plant kingdom glucose can also be substituted by other different sugars, such as galactose, rhamnose, arabinose, and xylose [49]. Increasing levels of glycosidation increases both the chemical stability and water/alcohol solubility of the anthocyanidins, but triglucosidic anthocyanins normally do not occur in grapes. 

Since grapes are rich sources of natural anthocyanins, grape anthocyanins (especially in *V. vinifera*, *V. labrusca*, *V. rotunfolia*, *V. rupestris*, and their hybrids) have been extensively studied [1,24,25,26,27,28,29]. In the *V. vinifera* grape varieties, the principal individual anthocyanins are 3-*O*-monoglucosides of delphinidin, cyanidin, petunidin, peonidin and malvidin, but not that of pelargonidin. In the non-*V. vinifera* grape varieties, 3-*O*-monoglucosides and 3,5-*O*-diglucosides of all the above anthocyanidins, even the pelargonidin, are also found. Recently, several literatures reported the existence of pelargonidin-3-*O*-glucoside at trace level in some *V. vinifera* cultivars [54,55]. Their absence in the previous studies seems not to result from their nonexistence, but rather from their typical extremely low concentration that is usually under the detection threshold. Nevertheless, individual anthocyanins are not particularly stable and are relatively easily oxidized, especially in grape products, and wines. A complex series of mechanisms can help to improve the color stability of anthocyanins, including short-term factors, such as self-association and copigmentation, and long-term factors, such as the formation of pyranoanthocyanins and polymeric anthocyanins [56,57,58,59,60].

### 2.2. Acetylated Anthocyanins

**Figure 2:**
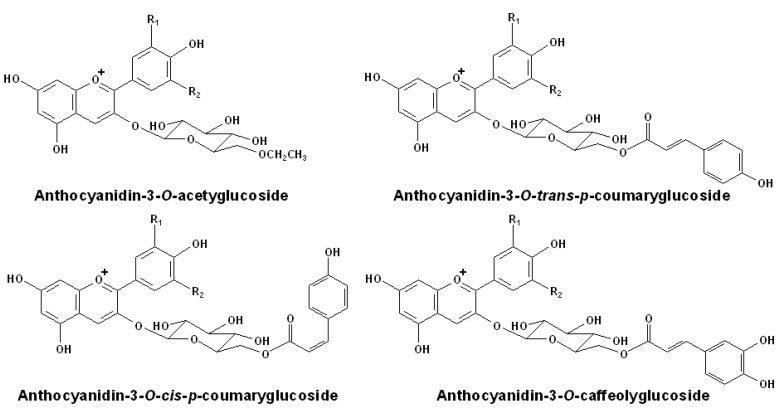
Structures of normal acetylated anthocyanins in grapes [61].

In addition, many anthocyanins have sugar residues which are acylated with aromatic (such as *p*-coumaric acid, caffeic acid, ferulic and sinapic acid) or aliphatic acids (such as acetic, malic, malonic, oxalic and succinic acid) at the C_6″_ position at the glucose moiety. The acylation of the sugar in the anthocyanins can also promote their chemical stability [62,63,64]. Interestingly, with *p*-coumaric acid, two stereoisomers of anthocyanidin-3-*O*-*p*-coumarylglucosides can be detected in grapes, which have the opposite directions of the *p*-coumaric acid moieties (*cis* or *trans* forms) [61]. The structures of normal acetylated anthocyanins found in grapes are illustrated in Figure 2.

However, not all of the red *V. vinifera* grapes can produce acetylated anthocyanins in their berry skin. For example, the widely grown cultivar Pinot Noir does not synthesize acetylated anthocyanins, and the other anthocyanins are also at relatively low levels [65]. And also, some red-colored mutants of white *V. vinifera* cultivars, such as red ‘Chardonnay’ and pink ‘Sultana’, also produce no acylated anthocyanins [66]. Furthermore, other grape species, such as *V. rotundifolia* (muscadine grapes) and *V. amurensis*, also accumulate no acylated anthocyanins in their berries [24,25]. All in all, the detailed information of the free anthocyanins that typically can be identified in grape berries is summarized in Table 1, as shown above.

**Table 1:** The chromatography and mass spectrometry information of free anthocyanins that are commonly found in various grape berries [35,36,67,68,69].

Compound	Molecular ion M^+^(m/z)	Fragment ion M(m/z)	Maxima absorptionwavelengths (nm)
Delphinidin-3-*O*-monoglucoside	465	303	280,523
Cyanidin-3-*O*-monoglucoside	449	287	279,515
Petunidin-3-*O*-monoglucoside	479	317	277,526
Peonidin-3-*O*-monoglucoside	463	301	279,515
Malvidin-3-*O*-monoglucoside	493	331	278,530
Pelargonidin-3-*O*-monoglucoside	433	271	505
Delphinidin-3-*O*-acetylglucoside	507	303,465	280,521
Cyanidin-3-*O*-acetylglucoside	491	287,449	279,514
Petunidin-3-*O*-acetylglucoside	521	317,479	280,530
Peonidin-3-*O*-acetylglucoside	505	301,463	280,518
Malvidin-3-*O*-acetylglucoside	535	331,493	280,521
Delphinidin-3-*O*-coumarylglucoside	611	303,465	282,530
Cyanidin-3-*O*-coumarylglucoside	595	287,449	283,522
Petunidin-3-*O*-coumarylglucoside	625	317,479	280,531
Peonidin-3-*O*-coumarylglucoside	609	301,463	279,523
Malvidin-3-*O*-coumarylglucoside	639	331,493	280,521
Peonidin-3-*O*-caffeoylglucoside	625	301,463	283,525
Petunidin-3-*O*-caffeoylglucoside	641	317,479	Unknown
Malvidin-3-*O*-caffeoylglucoside	655	331,493	283,538
Malvidin-3-*O*-feurlylglucoside	669	331,493	532
Delphinidin-3,5-*O*-diglucoside	627	303,465	282,520
Cyanidin-3,5-*O*-diglucoside	611	287,449	282,516
Petunidin-3,5-*O*-diglucoside	641	317,479	274,523
Peonidin-3,5-*O*-diglucoside	625	301,463	278,513
Malvidin-3,5-*O*-diglucoside	655	331,493	275,524
Pelargonidin-3,5-*O*-diglucoside	595	433,271	Unknown
Delphinidin-3-*O*-acetylglucoside-5-*O*-glucoside	669	303,465,507	Unknown
Cyanidin-3-*O*-acetylglucoside-5-*O*-glucoside	653	287,449,611	280,516
Petunidin-3-*O*-acetylglucoside-5-*O*-glucoside	683	317,479,641	280,530
Malvidin-3-*O*-acetylglucoside-5-*O*-glucoside	697	331,493,655,535	278,530
Delphinidin-3-*O*-coumarylglucoside-5-*O*-glucoside	773	303,465,627,611	279,530
Cyanidin-3-*O*-coumarylglucoside-5-*O*-glucoside	757	287,449,611,595	280,524
Petunidin-3-*O*-coumarylglucoside-5-*O*-glucoside	787	317,479,641,625	280,530
Peonidin-3-*O*-coumarylglucoside-5-*O*-glucoside	771	301,463,625,609	279,520
Malvidin-3-*O*-coumarylglucoside-5-*O*-glucoside	801	331,493,655,639	280,530
Delphinidin-3-*O*-feruloylglucoside-5-*O*-glucoside	803	303,465	Unknown
Petunidin-3-*O*-caffeoylglucoside-5-*O*-glucoside	803	317,479,641	Unknown

### 2.3. Anthocyanin Derived Pigments

**Figure 3:**
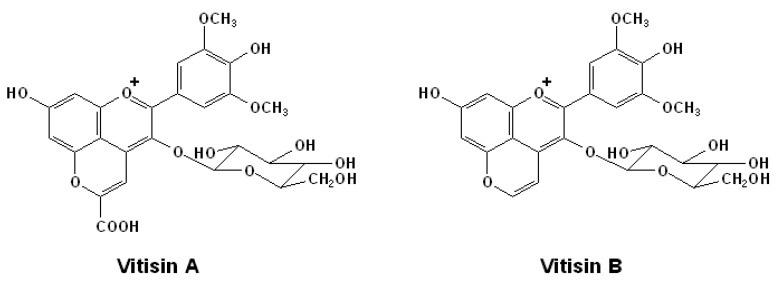
Structures of vitisin A and vitisin B [71].

It has been commonly assumed that anthocyanin derived pigments, such as pyranoanthocyanins, do not occurring in fresh grapes, but only in stored grapes and aged red wines. For example, the well known pyranoanthocyanin, vitisin A, is formed via the reaction between malvidin-3-*O*-monoglucoside and pyruvic acid, which is an important intermediate in the alcoholic fermentation [70]. Thus, typically vitisin A would not be expected to exist in fresh grapes. Similarly, vitisin B is the product of the reaction between malvidin-3-*O*-monoglucoside and acetaldehyde, which is the main product of ethanol oxidation [58,71]. However, the occurrence of vitisin B in Concord (*V. labrusca*), Salvador (*V. vinifera* × *V. rupestris*), and Rubired (*V. vinifera* × *V. rupestris*) grape juices that indicate that it might occur in the corresponding fresh grapes has been reported [69]. Figure 3, shows the structures of vitisin A and vitisin B.

## 3. Biosynthesis of Anthocyanins in Grapes

**Figure 4:**
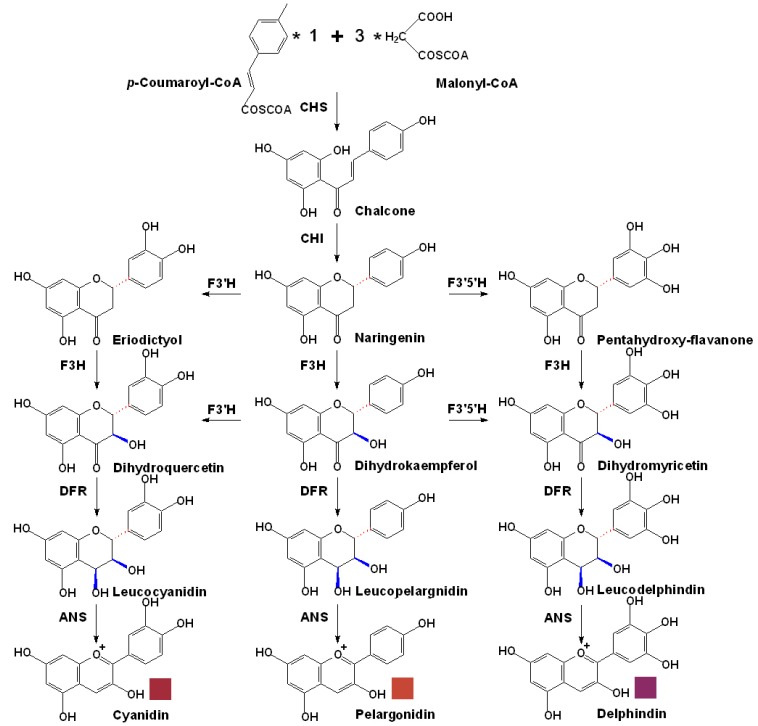
The basic upstream flavonoid pathway leading to the biosynthesis of colored anthocyanidins in grapes. **CHS**, chalcone synthase; **CHI**, chalcone isomerase; **F3H**, flavanone 3β-hydroxylase; **F3’H**, flavonoid 3’-hydroxylase; **F3’5’H**, flavonoid 3’,5’-hydroxylase; **DFR**, dihydroflavonol 4-reductase; **ANS**, anthocyanidin synthase [2,3,4,5,43,44,45,47].

Free anthocyanins in grape berries are synthesized via the flavonoid pathway, which shares the same upstream pathway with proanthocyanidins, until the formation of anthocyanidins by the catalysis of anthocyanidin synthase (ANS), also known as leucoanthocyanidin dioxygenase (LDOX) [2,3,4,5,45,72,73]. In the past, ANS used to be considered as the first key enzyme that could lead the flavonoid flux into the anthocyanin branch. However, recent work has revealed that ANS also plays a significant role in the biosynthesis of proanthocyanidins [43,44,45,47]. Its products, anthocyanidins are not only the direct substrates for the anthocyanin synthesis, but also can be catalyzed by anthocyanidin reductase (ANR) to produce (2*R*,3*R*)-*cis*-flavan-3-ols, which are a group important substrates for the proanthocyanidins synthesis [74,75]. Consequently the whole pathway can be divided into two parts, the basic flavonoid upstream pathway and the specific anthocyanin downstream branch, as shown in Figure 4.

### 3.1. The Basic Flavonoid Upstream Pathway of Anthocyanin Biosynthesis

The basic flavonoid upstream pathway has been investigated extensively at both the biochemical and genetic levels, which are not only restricted in some model plants, such as *Arabidopsis thaliana*, soybean or barley, but also in many other plants, especially in grapes [43,44,45,47]. Previous studies revealed that the enzymes acting earlier in the flavonoid pathway are usually encoded by larger gene families, whereas the enzymes acting late in the pathway are commonly encoded by one single active gene [76]. This may result from fact that the earlier-acting genes usually possess more diverse metabolic functions, which requires correspondingly diverse control of gene expressions by using gene duplication. In contrast, the later-acting genes normally point to simpler and more specific metabolic functions and the further duplication of these genes is usually unnecessary. However, this rule is not exactly correct in grape anthocyanin biosynthesis. Nevertheless, the early reports that related the genes encoding of the key enzymes of anthocyanin biosynthesis and their expression analysis in grapes occurred more than 12 years ago, and new information has been added in the following years [77,78,79].

Generally, by the action of chalcone synthase (CHS), three malonyl-CoA molecules and one *p*‑coumaroyl-CoA molecule can condense to produce a naringenin chalcone [76,80,81]. In grapes (*V. vinifera*), there are at least three genes encoding CHS, *Chs1* (AB015872), *Chs2* (AB066275), and *Chs3* (AB066274), which are transcribed under different controls [82,83]. However, their unanimous product, the naringenin chalcone is not only used in the synthesis of anthocyanins or proanthocyanidins, but also in the formation of other phenolic compounds. Thus, the three different CHSs may act in three different pathways to produce different secondary metabolites [80,82,83]. Furthermore, although the expressions of these *Chs* genes at both transcript and protein levels are relatively low, their high biosynthetic efficiency may also guarantee their high production [82]. 

Then, after the action of chalcone isomerase (also known as chalcone-flavanone isomerase, CHI), the naringenin chalcone can be modified to its isomer naringenin flavanone stereospecifically and quickly, which initially consists of the basic three rings of the general C6-C3-C6 flavonoid skeleton [1,84]. It is worth noting that the product of CHI is almost absolutely the biologically active (2*S*)-flavanone, which is crucial to the subsequent reactions. In grapes, by characterizing the grape transcriptome, a gene is encoding a putative CHI that is expressed strongly at the onset of veraison [85]. 

The B ring of the naringenin flavanone can be further hydroxylated by flavonoid 3’-hydroxylase (F3’H) or flavonoid 3’5’-hydroxylase (F3’5’H) to produce eriodictyol or pentahydroxyflavanone, respectively [86,87]. All of the three (2*S*)-flavanones can be modified by the catalysis of flavanone 3β-hydroxylase (F3H, also known as FHT) to produce the corresponding dihydroflavonols. Furthermore, the direct enzymatically oxidized product of naringenin flavanone, dihydrokaempferol, is also the potential substrate for F3’H and F3’5’H, to produce the corresponding dihydroflavonols, dihydro-quercetin and dihydromyricetin, respectively [76]. In grapes, there are at least two putative F3Hs (CAA53579 and P41090), the expressions of which are quite similar to that of the CHS (CAA53583) or ANR (CAD91911) [86]. Recently, four genes encoding F3’H, *F3’h1* (AB213602), *F3’h2* (AB213603), *F3’h3* (AB213604) and *F3’h4* (AB213605), and one gene encoding F3’5’H, *F3’5’h* (AB213606) were identified and characterized in grapes. Furthermore, the expression of the four *F3’h* genes are different from each other in various grape organs, and the expression of *F3’5’h* is higher than that of *F3’h* at the transcriptional level in grape skin of *V. vinifera* cultivars, such as Cabernet Sauvignon and Dornfelder, which explains their different anthocyanin composition. However, a similar test of berry skin of Muscat Hamburg obtained the opposite results [87]. In yet another report, published almost in the same period, besides the gene cloning of the putative *F3’h* (AJ880357) and *F3’5’h1* (AJ880356) in grapes, the results also revealed that the temporal and tissue-specific expression of *F3’h*, *F3’5’h1* and *CytoB5* (encoding a putative cytochrome *b_5_* homolog in grapes, TC45693) was coordinated with the accumulation of the respective hydroxylated flavonoids, such as the anthocyanins [88].

After these modifications, dihydroflavonol 4-reductase (DFR) can reduce these dihydroflavonols to their corresponding leucoanthocyanidins [76]. These reactions also have extremely high stereospecificity, the absolute configuration of their products was determined to be (2S,3S,4S). Interestingly, by inducing Pi deficiency and feeding of elevated dihydroquercetin in a novel cell suspension culture initiated from nonpigmented grape cells, anthocyanins were synthesized with the increased DFR activity [89]. However, leucoanthocyanidins besides being used to synthesize the corresponding anthocyanidins, they can also be reduced to their corresponding (2*R*,3*S*)-*trans*-flavan-3-ols by the action of leucoanthocyanidin reductase (LAR), which is the direct substrate for proanthocyanidins polymerization [90,91]. Furthermore, even the leucoanthocyanidins themselves are also considered as a class of potential substrates for proanthocyanidins biosynthesis [43,44,45]. Although DFR has been widely studied in numerous plants, until recently little has been known, especially in grapes, about its structural and biochemical properties. Recently, the crystal structure of the *V. vinifera* DFR was determined, and the crucial region of substrate binding and recognition was also confirmed [92]. Furthermore, the promoter of the gene encoding the grape DFR (CAA72420.1) was cloned, and analyzed. A specific sequence of the *Dfr* promoter might be involved in the expression of the gene in grape berries and white light, calcium and sucrose can induce the *Dfr* gene expression, suggesting a UV receptor signal transduction pathway might involved in the induction of the *Dfr* gene [93].

As mentioned above, ANS also plays a pivotal role in the biosynthesis of both anthocyanins and proanthocyanidins [43,44,45]. By the catalysis of ANS, with the help of ferrous iron, the colorless leucoanthocyanidins can be oxidized to their corresponding colored anthocyanidins. However, as a member of the 2-oxoglutarate-dependent oxygenase family, just like F3’H and flavonol synthase (FLS), ANS usually possesses multiple functions excepting the basic role mentioned above, for it usually catalyses the production of dihydroflavonols or flavonols [73,76]. Recently, the promoter of the grape *Ans* gene (CAA53580) was also cloned, and analyzed. Similar to that of *Dfr*, the *Ans* promoter also has several putative DNA binding motifs and can be induced by light, calcium and sucrose [73].

### 3.2. Specific Pathway for the Anthocyanin Modification

**Figure 5:**
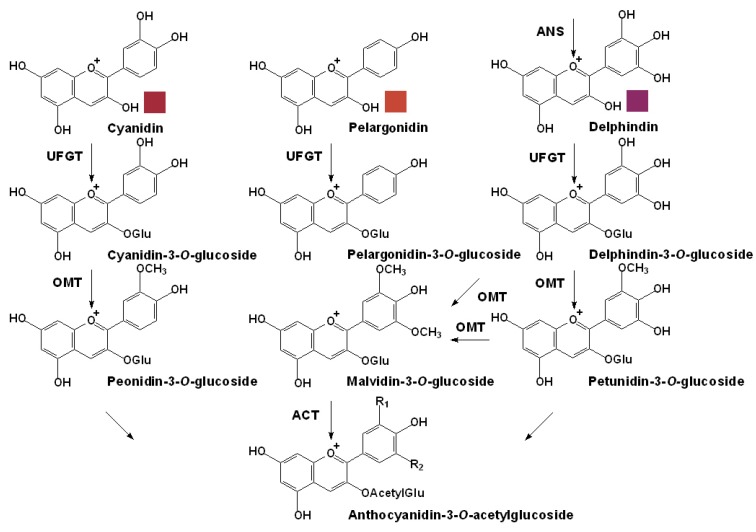
The specific pathway for the anthocyanin modification of free anthocyanidins in grapes. **UFGT**, flavonoid glucosyltransferase; **OMT**, *O*-methyltransferase; **ACT**, anthocyanin acyltransferase [51,72].

Consequent to the activity of ANS, colored anthocyanidins are formed, but their immediate modification, mainly by glycosylation, methylation and acylation is very necessary for their stabilization as vacuolar anthocyanins. This usually occurs in the cytosol just after the biosynthesis of the unstable anthocyanidins there, as shown in Figure 5 [72]. Mutants of red grapes lacking the expression of the genes for the initial glycosylation of anthocyanidins usually accumulate no anthocyanins in their berries, though the intact anthocyanidin biosynthetic pathway exists [94]. In the last decade by cloning the related genes in various plants our appreciation of the specific anthocyanin downstream branch has been advanced greatly.

Glycosylation is an important modification for increasing the hydrophilicity and stability of anthocyanins, because the anthocyanidins are inherently unstable under the physiological conditions. In plants, UDP-glucose: anthocyanidin: flavonoid glucosyltransferase (UFGT), especially the anthocyanidin/anthocyanin glycosyltransferases catalyze the *O*-glycosylation of anthocyanidins or anthocyanins, which recognize either anthocyanidins or anthocyanins as sugar acceptors and UDP-sugars as the sugar donors [51,76]. Several other sugar donors are also be used occasionally by grape UFGT, such as UDP-galactose, UDP-rhamnose, UDP-xylose, UDP-glucuronic acid and UDP-arabinose [72].

In *V. vinifera* grapes, anthocyanidins can only be O-glycosylated at the C3 position with the addition ofglucoses by the activity of UFGT [51]. Thus, *V. vinifera* UFGT used to be called as 3-*O*-glucosyltransferase (3-GT) for short. Normally, the *Ufgt* expression is only detected in berry skin after the onset of veraison specifically, whereas most of the upstream genes may express constitutively in different organs and tissues at diverse levels [77,78,79]. The UFGT enzyme that has been isolated from *V. vinifera* cell suspension cultures shows highest activity with cyanidin as acceptor, but it can also use delphinidin greatly as well as pelargonidin, peonidin, petunidin, and malvidin at lower levels at its optimal pH 8.0 [95]. Furthermore, there are no differences in the *Ufgt* gene coding and promoter sequences between white grapes and their red skin sports, but *Ufgt* gene only expresses in the red sports. This result revealed the significant role of UFGT in the anthocyanin biosynthesis and suggested that its expression is under extremely strict regulation of some genetic transcriptional factors [96]. Its expression has high organ and time specificity, as well as high substrate specificity [97].

Here, it is worth discussing the poor production and accumulation of pelargonidin-3-*O*-glucoside in common *V. vinifera* grape berries, which usually results in its ‘absence’ in their anthocyanin profiles’ analysis [1,33,65]. In grapes, the biosynthetic pathway for pelargonidin-3-*O*-glucoside is intact, and the relatively wide substrate specificity of the structure genes, for example CHI, F3H, DFR, ANS and UFGT, cannot get rid of the possibility of its existence [76,77,78,79]. However, our recent research supported the evidence of the presence of pelargonidin-3-*O*-glucoside in grapes at trace levels [55]. So, what causes the extremely low concentration of pelargonidin-3-*O*-glucoside in grape berries? The comparatively higher activity of F3’H and F3’5’H in grapes may explain this question. In this pathway, most of the naringenin and dihydrokaempferol (the precursors of pelargonidin) were used to form the flavonoids with more than one hydroxyl group, and the following products are used in the formation of corresponding anthocyanins or flavonols [87,88]. As a result, there is little flux pointing to the production of pelargonidin anthocyanins or kaempferol glycosides. Thus, the major flux is pumped to the ‘side path; not the ‘central way’ that leads to the synthesis of pelargonidin-3-*O*-glucoside. This assumption can be reasonably explained by the analysis of anthocyanin or flavonol profiles in many *V. vinifera* varieties [1,33,65].

In most non-*V. vinifera* grapes or their hybirds, *O*-glycosylation can also occur at both of the C_3_ and C_5_ positions to form anthocyanidin-3,5-*O*-diglucoside, while only trace amount of 3,5-*O*-diglucoside can be found in some European cultivars [1,24,25,26,27,28,29]. A recent research paper reported that the cloning of the non-functional allele of the functional 5-*O*-glucosyltransferase (5-GT) from the heterozygous hybrid cultivar. Comparison and analysis of the sequences revealed that two mutations of this gene inactivated its 5GT function in *V. vinifera* grapes and resulted in the absence of anthocyanidin-3,5-*O*-diglucosides [52].

In the plant kingdom, it was reported that almost 90% of the anthocyanins were modified by methylation of the six common anthocyanidins [49]. Of these three methylated anthocyanidins, peonidin, petunidin and malvidin, comprise 20% of the total reported anthocyanidins [1]. With the participation of *S*-adenosyl-L-methionine (SAM), *O*-methyltransferase (OMT, also known as anthocyanin *O*-methyltransferase AOMT), can mediate the methylation of the hydroxyl groups at the C_3’_ positions or both at the C_3’_ or C_5’_ positions on the B rings of the anthocyanins [98]. In grapes, for significant OMT activity on anthocyanins requires the presence of divalent cations such as Mg^2+^, and belongs to the Class I OMTs. The hydroxyl group at the C_4’_ position on the B ring of the anthocyanins is seldom methylated. In a much earlier report, a highly specific OMT was partially purified and characterized from a cell suspension of *V. vinifera* L. cv. Gamay Fréaux. This was found to almost absolutely catalyze the transfer of the methyl group of SAM to the hydroxyl group at the C_3’_ position of cyanidin-3-*O*-glucoside, but to have a low affinity for cyanidin and did not methylate either cyanidin-3-*O*-*p*-coumaroylglucoside or delphinidin [99]. Interestingly, a recently found novel divalent cation dependent OMT from grapevine seems to have lower substrate specificity. It can mediate the methylation of both anthocyanins and flavonols and prefers 3^’^,5^’^ methylation when 3^’^,4^’^,5^’^ hydroxylated substrates are available. Further studies indicated that it is located in cytoplasm, and had the highest expression at veraison, indicating it plays crucial role in biosynthesis of anthocyanins in grape berries [100].

Acylation is one of the most common modifications of plant phenolics, including anthocyanins, resulting in greatly increased structural diversity of anthocyanins from the addition of aromatic and/or aliphatic constituents linked to the C6’’ positions of the glucosyl groups [1]. Anthocyanins, without the protection of acylation can be easily and quickly decolorized in neutral or weakly acidic aqueous solutions. Besides its beneficial effect on color stabilization, acylation can also promote color intensity for the anthocyanins, for the stacking of the polyphenolic moieties of the acylated anthocyanins can further stabilize the color of anthocyanins and may play an important role in the blue color shift [101]. The acylation of anthocyanins in plants are catalyzed by the action of anthocyanin acyltransferases (ACT, also known as AAT), which have really high substrate specificity, for both the anthocyanin acceptors and the acyl group donors. In plants, there are mainly two types of ACTs that are classified based on the acyl group donors: the BAHD family using acyl-CoA and the serine carboxypeptidase-like (SCPL) group using acyl-activated sugars [102]. Although in some important *V. vinifera* cultivars the acylated anthocyanins can account for more than 60% of the total anthocyanin content, until now there has been no report about the exact genes required for ACTs in grapes [1]. 

As shown above, anthocyanins are synthesized by an extremely complex network of all the structural enzymes in the pathway. However, color variation of the grape berries accord with the particular pattern of genotype-specific expression of the whole set of genes involved in anthocyanin biosynthesis in a direct transcript-metabolite-phenotype relationship. For an efficient production of anthocyanins in different steps, it is speculated that all of the key enzymes involved in the anthocyanin biosynthesis are associated with each other to form a multi-enzyme complex [2,3,4,5,72]. Published evidence indicates the existence of such complex in some model plants, such as *A*. *thaliana* [72]. However, to exactly understand the working mechanism of the entire complex in *vivo* still needs much more work, especially in the case of grapes.

### 3.3. Transportation and Localization of Anthocyanins

The distribution of anthocyanins in the different branches or a whole grape bunch is highly variable, depending on many physiological and climatic factors [103]. For the majority of red grape cultivars, such as Cabernet Sauvignon and Pinot Noir, the anthocyanins accumulate mainly in the hypodermal cell layers of the berry skin after veraison [1,77,78,79]. However, there are still a few special grapes of *V. vinifera* cultivars or their hybrids that can also accumulate high content of anthocyanins in their pulp. For example, Garnacha Tintorera, also named Alicante Bouschet, a teinturier (or dyed) cultivar with red skin and flesh after ripening, is a hybrid of Grenache and Petit Bouschet [54].

Although in grape cells, all of the structural genes involved in the anthocyanin biosynthesis are located on the endoplasmic reticulum membranes or in the cytoplasm, where the anthocyanins are directly produced, almost all of the anthocyanins accumulate in the vacuoles [104,105,106]. Thus, the effective intracellular transport of anthocyanins from their synthesis site to their storage site is a crucial problem. While the biosynthesis of anthocyanins has been thoroughly investigated, only a little is known about the exact mechanism of anthocyanin transportation. Several types of transporters have been proposed in grapes [107,108,109,110,111,112,113,114,115,116,117,118]. 

It has been suggested that the anthocyanins could be transported into the vacuoles via the non-covalent activity of glutathione *S*-transferase (GST) [107]. The vacuole accumulating anthocyanins used to be named as anthocyanic vacuolar inclusion (AVI), which is a non-membrane body containing a complex mix of huge dense of anthocyanins, proanthocyanidins and other compounds. Several genes for GSTs with such functions have been cloned and identified in plants. These include the *TT19* gene (encoding a type I GST) of *A*. *thaliana*, the *Bronze-2* gene (encoding a type III GST) of maize and the *AN9* gene (encoding a type I GST) of petunia, which are all the members of the *Gst* gene family [109,110,111]. Although they encode different types of GSTs, they are all required for transferring anthocyanins into vacuoles. Definitely, these GST proteins can not transport anthocyanins themselves, they need to work with the glutathione *S*-conjugate pump, which is an ATP-binding cassette (ABC) transporter and belongs to the multidrug resistance-associated protein (MRP) family [72]. In *A*. *thaliana* and maize, such specific MRPs involved in anthocyanin transportation have been characterized [112]. 

Interestingly, several *Gst* genes were identified in grapes, which were recognized as putative anthocyanin transporters. In one study of four specific co-expressed genes with anthocyanin accumulation in grape berries, one was identified as a gene encoding a type I GST protein required for vacuolar transport of anthocyanins, which is orthologous to the *AN9* gene [83]. In a more recent investigated, five GST proteins were purified from *V. vinifera* L. cv. Gamay Fréaux cell suspension, and two of them were characterized as anthocyanin transporters [113]. However, no MRP has been identified in grapes until now.

On the other hand, multidrug and toxic compound extrusion (MATE) transporters are another class of putative transport factors that may be involved in the intracellular transportation of anthocyanins. The *A*. *thaliana TT12* gene encodes a protein with 12 transmembrane segments exhibiting similarity to prokaryotic and eukaryotic secondary MATE protein, which is finally demonstrated to act as a vacuolar flavonoid/H^+^-antiporter on the vacuolar membrane. It can mediate the MgATP-dependent transport of cyanidin-3-*O*-glucoside in *vitro* [114]. Recently, two genes encoding MATE proteins were identified as anthocyanin transporter candidates in grapes. Both of the two genes were specifically expressed in fruit, and were concomitant with the accumulation of anthocyanins. Furthermore, both of the two expressed proteins were primarily localized to the tonoplast, and could mediate the MgATP-dependent and H^+^-dependent vacuolar transport of acylated anthocyanins. However, they could not transport the individual anthocyanins, such as malvidin-3-*O*-monoglucoside or cyanidin-3-*O*-mono-glucoside, indicating its substrate specificity and the importance of acylation for their uptake [115].

Besides the MRP and MATE transporters, there are some putative mechanisms for anthocyanin transportation, such as the pH-dependent transporters and vacuolar protein (VP), which have been found in several plants, but not in grapes [116,117]. A putative flavonoid translocator, which may be responsible for the transport of anthocyanins, and which is similar to mammalian bilitranslocase, has recently been found in ripening berries of *V. vinifera* L. cv. Merlot [118].

### 3.4. Degradation of Anthocyanins

The individual anthocyanins are not particularly stable, and are susceptible to degradation. This is, affected by numerous factors, such as pH, temperature, structure, light, oxygen, solvents, enzymes and metallic ions [119].

Normally, *O*-glycosylation can stabilize the anthocyanins, which can be stored in vacuole without hydrolysis by anthocyanase. But anthocyanins are readily hydrolyzed to their corresponding anthocyanidins and sugars when the grape cells are infected or broken. Then, anthocyanins or anthocyanidins possessing *o*-diphenols structures are easily degraded by enzymatic and nonenzymatic oxidation. In the autoxidation of *o*-diphenol, besides the corresponding *o*-diquinone, hydrogen peroxide can also be formed as a by-product, which can participate in the further oxidation of other phenolic compounds [48]. 

**Figure 6:**
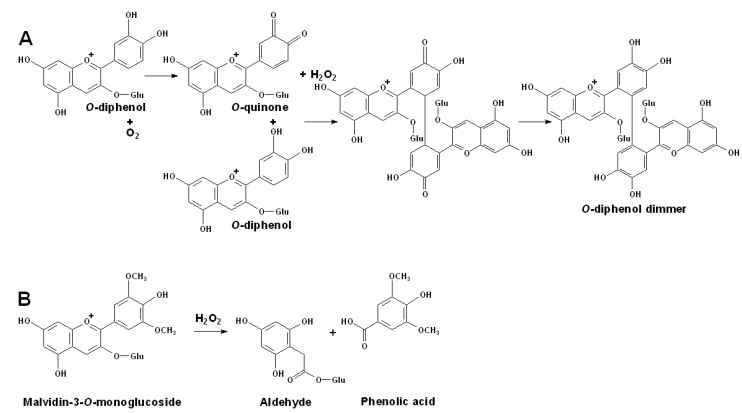
**(A)** Autoxidation of *o*-diphenols to produce *o*-diquinones and the following *o*-diphenols dimer. **(B)** Degradation of anthocyanin by the oxidation of hydrogen peroxide [48,120].

On one hand, the produced *o*-diquinones and hydrogen peroxides can be involved in the further oxidation of other *o*-diphenols to form dimeric *o*-diphenols, as shown in Figure 6A [48]. On the other hand, the hydrogen peroxide by-product can oxidize anthocyanins or anthocyanidins to the corresponding phenolic acid and aldehyde, as shown in Figure 6B [120].

However, the degradation does not usually occur in intact healthy grape berries. It only happens when the grape damaged by pathogen attack or physical disturbance.

## 4. Regulation of Anthocyanin Biosynthesis in Grapes

Based on color, grapes can be easily divided into two groups, red and white. The red cultivars can synthesize and accumulate anthocyanins in their berry skin to express their color, whereas the white cultivars normally do not produce any anthocyanins in their fruits. However, in red grapes, anthocyanin accumulation is often influenced by a series of factors, such as genetic regulation, phytohormone, and the viticultural environment.

### 4.1. Genetic Regulation of Anthocyanin Biosynthesis

As the secondary metabolites of the flavonoid pathway, anthocyanins are synthesized under the complex regulation of multiple regulatory genes at the transcriptional level [2,3,4,5,72]. Generally, the basic flavonoid upstream pathway of anthocyanin biosynthesis (down to the synthesis of anthocyanidins) is under the control of several different families of regulatory genes in plants, such as Myb transcriptional factors, Myc transcriptional factors (encoding basic helix–loop-helix proteins, bHLH) and WD40-like proteins, which also play crucial roles in the regulation of other flavonoid products, such as flavonols and proanthocyanidins [43,44,45,121]. By using several mutants of the model plant *A*. *thaliana* with low proanthocyanidin levels or low levels of both anthocyanins and proanthocyanidins, it has been possible to identify and characterize most regulatory genes of these categories including *PAP1 & PAP2* (Myb family), *GL3 & EGL3* (Myc family) and *TTG1* (WD40 family). These are connected to form a complex in a hierarchical network to regulate the expression of the structural genes involved in the flavonoid synthesis, such as *Chi*, *Chs*, *F3h,* and *Dfr* [121,122,123,124]. However, a series of Myb and Myc factors, such as MYBL2, MYB4 and BHLH32, are also found to work as negative regulators of the biosynthetic pathway of anthocyanins in *A*. *thaliana* [125,126,127]. 

In grapes, a series of R2R3-Myb transcriptional factors have been demonstrated to be involved in the control of different branches of the phenylpropanoid pathway, such as those for anthocyanins, flavonols, proanthocyanidins and lignins. The newly cloned genes of the transcriptional factors, *Vvmyb5a* and *Vvmyb5b* belong to this group [128,129]. However, some other MYB factors such as VvmybPA1 and VvmyPA2 specifically regulate the synthesis of proanthocyanidins, but not anthocyanins, by controlling their expression, [130,131]. Although several transcriptional factors of Myc family have been found in grapes, no detailed information yet points to their relationship with anthocyanin synthesis, as well as the WD40-like proteins.

Moreover, the specific downstream branches leading to anthocyanin formation through glycosylation and subsequent modification (methylation and acylation) are under the specific control of several regulatory factors. Until now, only several R2R3-Myb related transcriptional factors have been identified as specifically regulating anthocyanin accumulation [132]. The first breakthrough was the identification and characterization of the VlmybA1-1, VlmybA1-2 and VlmybA2 transcriptional factors in the *V. labrusca* grapes, which greatly facilitated the investigation of the regulation of anthocyanin biosynthesis in *V. vinifera* grapes [133,134,135].

In red *V. vinifera* grapes, such factors as the functional *VvmybA1* (*VvmybA1b* allele, AB111101), are only expressed after veraison and regulate anthocyanin biosynthesis during ripening by strict control of the expression of specific anthocyanin biosynthesis genes, especially UFGT, which plays a crucial role in the synthesis and accumulation of anthocyanins. Interestingly, in white *V. vinifera* grapes, a retrotransposon, *Gret1*, is inserted in the 5'-flanking region of its related non-functional *VvmybA1* gene (*VvmybA1a* allele, AB111101), resulting in the function losing its transcriptional factor [136,137]. In red skinned sports of white cultivars, the *Gret1* insertion is missing from their *VvmybA1* genes, leaving behind a solo long terminal repeat (LTR) as the functional regulatory gene. Thus, the bud mutations from white to red skins are caused by deletion of the inception region of the retrotransposon, *Gret1* [138]. Further studies have found that *VvmybA1* is not expressed in the young leaves, tendrils or stem epidermis of grapes that are normally colored, such as Cabernet Sauvignon, Dornfelder, or Muscat Hamburg or Muscat Bailey A, but only in berry skin, suggesting it is transcriptional tissue specific. However, it is transcribed in all the organs that accumulate anthocyanins in the teinturier cultivar Bailey Alicant A, indicating reduced organ specificity of expression in such cultivars [139]. Whatever, VvmybA1 factor is considered as the major gene determining anthocyanin synthesis, as well as the grape skin color [140].

Besides *VvmybA1*, two other families of *VvmybA* regulator genes from grapes, *VvmybA2* and *VvmybA3*, which have different multiple sequences, have also been cloned and identified [138]. The two similar genes, *VvmybA1* and *VvmybA2* are reported to be located on a single bacterial artificial chromosome and each of them can regulate the color accumulation of the grape berries. However, the *VvmybA2* allele in white grapes is inactivated by two non-conservative mutations. Sequence analysis of the *VvmybA2* alleles in 55 white cultivars suggests that the rare mutations occurring in the two adjacent regulatory genes are essential for the genesis of white grape cultivars and all of the extant white cultivars of grape vines have a common origin [141]. However, in several previous studies, just introducing *VvmybA2* may not induce the anthocyanin synthesis in white grape embryo. And also, it is speculated that all white grape cultivars have arisen from their different corresponding red cultivars by independent mutations. For example, the white cultivar Pinot Blanc is thought to be a white-skinned mutant of its corresponding red cultivar Pinot Noir resulting from loss of the functional *VvmybA1c-PN* allele (AB242302) [142]. Until now, no reasonable explanation accommodates these different opinions. More recent research about the quantitative genetic basis of anthocyanin variation of different colored grapes, ranging from white to the darkest purple, also concludes that the continuous variation in anthocyanin content in grapes can be explained mainly by the actions of a single gene cluster of three *VvmybA* genes (*VvmybA1*, *VvmybA2* and *VvmybA3*) [143].

### 4.2. Influence of Phytohormones on Anthocyanin Accumulation

Phytohormones, also known as plant hormones, regulate plant growth. Although usually produced in extremely low concentrations, phytohormones can regulate many aspects of plant growth [144]. Application of exogenous phytohormones can greatly modify the expression of the genes involved in the anthocyanin biosynthesis, as well as the production and accumulation of anthocyanins in grape berries. However, the signal transduction pathways of these phytohormones effecting the expression of these genes have not been identified until now. 

Abscisic acid (ABA) is a phytohormone involved in stress responses, especially in response to water stress, for ABA levels usually increase with growth restriction caused by water stress. Furthermore, ABA can also enhance dry matter accumulation in plant organs as a consequence of tissue relief from water stress. As early as the 1980s, it was reported that ABA could increase the anthocyanin content in grape skin and application of ABA might greatly enhance the color property of grapes [145]. Addition of ABA to the grape cell suspension culture also demonstrated that ABA can greatly promote anthocyanin production and the activity of CHI, a crucial enzyme in the flavonoid pathway for anthocyanin biosynthesis [146]. Further case studies indicated that ABA not only increased the anthocyanin content in the grape skin but also the expression of *Chs*, *Chi*, *Dfr* and *Ufgt* genes in the anthocyanin biosynthesis pathway, and the regulatory factors *VvmybA1* [147,148]. Recently it has been suggested that ABA spray could significantly increase the anthocyanin yield without negatively affecting the quality of the harvested grapes [149].

Ethylene acts as a plant hormone regulating many aspects of fruit ripening. Application of the ethylene-releasing compound, 2-chloroethylphosphonic acid (2-CEPA) can hasten the accumulation of anthocyanins in grape skin. In one recent investigation, treatment of grape berries with 2-CEPA stimulated the long-term expression of *Chs*, *F3h*, *Ans* and *Ufgt* (but not *Dfr*) and the related anthocyanin synthesis [150]. Additionally the post-harvest application of ethylene and /or 1-methylcyclopropene (1-MCP) to grape berries improved the stability of anthocyanins during storage [151].

Jasmonic acid (JA), derived from the fatty acid linolenic acid, is a member of the jasmonate class of phytohormones. JA and its analogs methyl jasmonate (MeJA) are usually produced by plants in response to many biotic and abiotic stresses. It was also reported that application of JA on grape cell suspension cultures irradiated with light increased anthocyanin production [152]. Besides, application of MeJA in combination with carbohydrates to grape cell suspension cultures stimulated the gene expression of *Chs* and *Ufgt*, resulting in anthocyanin biosynthesis enhancement [153].

Some other phytohormones, such as salicylic acid can induce the expression of the genes encoding phenylalanine ammonia-lyase (PAL) in grape berries, which is a crucial enzyme for phenylpropanoid metabolism, catalyzing the formation of *trans*-cinnamic acid. Thus, it may indirectly stimulate anthocyanin biosynthesis [154]. However, some phytohormones reportedly have a negative influence on anthocyanin accumulation in grape berries. For example, application of 2,4-dichlorophenoxyacetic acid (2,4-D) or 1-naphthaleneacetic acid (1-NAA) to grape berries suppresses the expression of anthocyanin biosynthesis related genes, as well as the regulatory factor *VvmybA1* and inhibits the accumulation of anthocyanins [147,148]. 

### 4.3. Influence of Non-hormone Chemicals of Anthocyanin Biosynthesis

Besides phytohormones, there are still some other endogenous and exogenous chemical factors that can regulate the anthocyanin biosynthesis in grapes. Although sugars are mainly accumulated in the pulp, the total sugar content in berry skin also increases during grape ripening. It has been reported that anthocyanins usually accumulate one week later than the massive increasing of the sugar content, which meant that the sugars in the skins had closed relationship with the anthocyanin biosynthesis [155]. However, there are two controversial opinions about this relationship. Some suggest that the sugars in the skins play a role as regulators in the synthesis of anthocyanins, while others state that sugars are important only as substrates for anthocyanin formation [155]. Regardless, most of the arguments are only restricted in the analysis of relationship between the accumulation of sugars and anthocyanins at the metabolite level. It has been recently reported that sugars can enhance the expression of the *F3h* gene and the accumulation of anthocyanins, raising new evidence for the first argument [156].

Ethanol in low levels can also stimulate the maturation of some fruits. Recent research revealed that 5% ethanol can enhance anthocyanin accumulation in grape berries at veraison. Further research indicated that low level ethanol can inhibit the expression of *Cha*, *F3h*, *Dfr* and *Ans*, while greatly enhancing the expression of *Ufgt*. These results highlight the significant role of UFGT in anthocyanin biosynthesis, and also suggest that low level ethanol can trigger grape expression leading to anthocyanin accumulation during ripening [157].

Stilbenes such as resveratrol, are usually accumulated in fruit berries very rapidly when healthy grape berries are exposured to potential pathogen attack or UV irradiation [158]. However, synthesis of resveratrols usually decreases rapidly at veraison, as anthocyanins accumulate rapidly in berry skin, suggesting resveratrol and anthocyanins may compete with each other for the potential substrates. Further studies demonstrated that CHS may compete with resveratrol synthase (RS) after the onset of fruit ripening, indicating that action of resveratrol as a phytoalexin may suspend the anthocyanin synthesis, which can be easily found in the diseased grape berries [159]. 

However, some exogenous chemicals can also regulate the biosynthesis of anthocyanins in grape berries. Eutypine [4-hydroxy-3-(3-methyl-3-butene-1-ynyl)benzaldehyde], is a toxin produced by *Eutypa lata*, the pathogen causing *Eutypa* dieback in grapevines. Although this toxin does not reduce the expression of several structural genes involved in anthocyanin synthesis, including *Chs*, *F3h*, *Dfr* and *Ans*, it can dramatically reduce the expression of *Ufgt* and inhibit the accumulation of anthocyanins in grapes [160].

### 4.4. Influence of Cultivating Conditions on the Anthocyanin Biosynthesis in Grape Cell Suspension

Anthocyanins are increasingly being used as natural colorants in the food industry and red grapes are a common source [23]. Therefore, a lot of research has been focused the improvement of anthocyanin production by grape cell cultures [161].

Most of the grape cell cultures investigated for the production of anthocyanins come from two main sources, the teinturier cultivars Gamay Freaux or Bailey Alicant A. In most instances, these cultures can be induced to produce anthocyanins by continuous light exposure. There are some other derivatives which can synthesize under low light or even in dark [162,163]. Whatever, anthocyanin production is dramatically influenced by cultivation conditions, especially by metabolic or osmotic stresses [164].

In intact plants, water stress can greatly enhance secondary metabolism, particularly biosynthesis of flavonoids such as anthocyanin. A similar response also exists in grape cell suspension cultures. Regulation of the osmotic potential of culture medium may be useful in controlling anthocyanin production [164]. Elevating the sucrose content increases the external osmotic potential and hence can enhance the biosynthesis of anthocyanins. Similar improvements can also be achieved by adding mannitol to the culture medium. The reduction of nitrate to a critical level can also enhance the anthocyanin production by removing an inhibitory effect to anthocyanin production and transportation [165]. In contrast, high ammonium concentrations in the medium can reduce and inhibit the accumulation of anthocyanin [166]. Combined, the effect of high sugar and low nitrogen in the medium can maximize the anthocyanin production in grape cell suspension cultures [165]. Additionally, phosphate deprivation can enhance the DFR activity significantly and correspondingly increase anthocyanin accumulation [167].

Interestingly, adding one supplement named carboxymethyl cellulose (CMC) to an N-medium of the grape cell cultures significantly enhances the production of anthocyanins. It was proposed that increasing in medium viscosity caused by the CMC reduces the hydrodynamic stress on the grape cells and enables the grape cells to grow in bigger size and produce more anthocyanins [168]. In further studies, application of air flow to the grape cell suspension cultures growing in such viscous additive further improved their anthocyanin accumulation [169].

There are still a lot of other cultivation factors, such as phytohormones besides resveratrol, pH of the medium, light exposure, physical parameters such as temperature that influence anthocyanin production of the grape cell suspension culture [161]. 

### 4.5. Environmental Influence on Anthocyanin Biosynthesis of Grapevine

As discussed above, anthocyanin biosynthesis is usually influenced by a series of environmental factors, such as sunlight exposure, UV irradiation, temperature, water, and so on. These can significantly modify the content and the composition of the grape berry anthocyanins by affecting both the expression of the structural and regulatory genes [1].

Unlike some fruits that have an absolute light requirement for anthocyanin synthesis in their skins, grapes can accumulate anthocyanins in their skins with or without light. Although several studies revealed that light exposure has some positive effects on cluster anthocyanin concentration, exclusive shading can reduce the mRNA accumulation of the transcriptional factor VvmybA1 to a greater or lesser extent, and the expression of several structural genes in anthocyanin biosynthesis, such as *Chs* and *F3h.* Intense sunlight caused excessive sunburn in exposed berries and reduced the anthocyanin accumulation, and the associated high temperature can also inhibit the color development. In some other studies, involving fruit-zone leaf removal the increased sunlight exposure caused sunburn damage and reduced anthocyanin accumulation [147,170]. Thus, for the maximum production of anthocyanins in grape berries, moderate sunlight exposure is necessary, but the extent varies among different cultivars. 

It has been demonstrated in numerous plants, that UV irradiation can stimulate the expression of the genes involved in the anthocyanin biosynthesis and hence result in the enhancement of anthocyanin accumulation [171]. Until now, only few publications or papers have referred to the relationship between UV irradiation and anthocyanin biosynthesis in grape berries. In studies of the promoters of the grape *Dfr* and *Ans* genes, the results revealed that they could be induced via the UV-A/blue receptor signal transduction pathway [73,93]. In a case study, increasing the intensity of white light and/or UV-A exposure was found to enhance the anthocyanin accumulation in Gros Colman grapes [172].

As well as light exposure, temperature is another important environmental factor that influences anthocyanin synthesis [173]. Generally, low temperatures, such as 25 °C, favor the anthocyanin biosynthesis, whereas high temperatures, such as 35 °C are associated with anthocyanin degradation and inhibition of anthocyanin accumulation. In grapes, high night temperatures inhibit the gene expression of *Chs*, *F3h*, *Dfr*, *Ans* and *Ufgt* at the early stages of ripening and dramatically reduce the activity of UFGT, resulting in poor production of anthocyanins [174,175]. With the help of labeled C^13^ molecules, it was found that the C^13^-labelled anthocyanins produced before high temperature exposure were markedly reduced after treatment, suggesting that level was not only influenced by the lower expression of the structure and regulatory genes, but also by the degradation of the previously synthesized anthocyanins [176]. From a macroscopic view, it is easy to state that the lowest concentration of anthocyanins in the berries is usually obtained in the warmest year, whereas in the cooler year the grapes will produce more anthocyanins [177,178]. However, in research involving different treatments of harvest grapes, some interesting and consistent results have been found. Low storage temperatures induced the accumulation of anthocyanins and the mRNA of related structure genes, whereas simultaneous high CO_2_ treatment lessened the gene expression and anthocyanin accumulation [179,180].

Water status is the third important environmental factor that can influence anthocyanin biosynthesis. During ripening, under water deficit conditions, anthocyanin biosynthesis can be greatly stimulated resulting in enhanced anthocyanin accumulation. Drought conditions berry dehydration can further concentrate the anthocyanin content in the berries. Besides the general biosynthetic genes, such as *Chs*, *F3h* and *Ufgt*, the genes related to the modification of the B ring, including *F3’5’h* and *Omt* could also be stimulated in grape berries on vines experiencing water deficit during ripening. This would enrich the production of the related hydroxylated and methoxylated anthocyanins [181]. Furthermore, water deficit might also result in the earlier expression of such biosynthetic genes and limit the expression of the structural genes involved in the synthesis of proanthocyanidins and flavonols and their accumulation [182]. However, there were conflicting results in a two year investigation, in which it was found that irrigation during ripening enhanced the content of several anthocyanins at harvest, as well as berry weight and crop yield [183]. 

### 4.6. Influence of Viticulture Practice on Anthocyanin Accumulation of Grapevine

As crucial pigments in red grapes, anthocyanins also play significant roles in the color of their main product, red wine. Therefore, considerable attention has been paid to viticultural practices used by growers in order to promote anthocyanin accumulation in grape berries. However, the variation of anthocyanin composition and concentration in grapes is so complicated and varied as a consequence of the many viticultural factors, such as cultivar, climate, seasonal influences, canopy management, fertilizer and water regimes and so on [1].

Cluster thinning subsequent to fruit setting can help regulate yield and improve fruit composition at harvest. Both mechanical and hand thinning practices used to reduce the bunch numbers, can advance fruit maturity, increase bunch and berry weights, enhance the anthocyanin accumulation and improve fruit quality [184]. In contrast, cluster thinning at veraison has a minimal effect on ripening time and the weight of grape skins, whereas it results in a lower total acidity and slightly higher pH, as well as a higher concentration in berry skin [185].

Comparison of different viticulture patterns also offers interesting results. The differences between anthocyanins of the grapes produced in the high-quality and low-quality vineyards are more obvious in terms of concentration than in terms of composition. In some cultivars, such as Tempranillo and Cabernet Sauvignon, high-quality grapes had larger anthocyanin content than that of the low-quality ones. But in other cultivars, such as Garnacha, the obtained result is the opposite, suggesting that the cultivar specificity is more predominant than the quality of the vineyards [186]. Other comparisons between the anthocyanin content of the grapes produced in organic or biodynamic vineyards and conventional ones achieve a consistent answer, which is the latter one can accumulate significantly higher anthocyanin than the former one [187]. Furthermore, soil modifications also affect the anthocyanin accumulation [188].

Some other studies investigated the influences of the grapevine vigor on the anthocyanin composition and content in the harvest grapes, even in their corresponding wines [189,190]. Research has been conducted in various aspects, such as the effects of girding, virus and attack by other diseases, usage of fertilizer, application of partial root zone drying (PRD) irrigation technique, and so on [191,192,193].

## 5. Conclusions

Due to their biological specificity and the considerable commercial values, grapes of both *V. vinifera* and non-*V. vinifera* cultivars continually attract attention of researchers in related fields. With the help of modern technology, great improvements have been obtained in the investigation of red grape anthocyanin biosynthesis and regulation. Numerous anthocyanins of various structures have been identified in grape berries. Diverse biosynthetic genes involved in anthocyanin production and transportation have been characterized. A series of transcriptional factors of Myb family related to specific regulation of anthocyanin synthesis in grape berries have been found. Yearly the information on the regulation of phytohormones, environmental factors and viticulture practices on the anthocyanin accumulation is increasing.

However, there are still many facets to the biosynthesis and regulation of anthocyanins in grape berries that are still unclear, such as the exact mechanism of anthocyanin intracellular transportation, the particular mechanism that regulates the period specific accumulation of anthocyanin in grape berries, the relationship of genetic control between anthocyanins and proanthocyanidins, and so on. We believe such questions will be revealed through continuing investigations in future decades.
